# In Situ Quantitative Measurement of HER2mRNA Predicts Benefit from Trastuzumab-Containing Chemotherapy in a Cohort of Metastatic Breast Cancer Patients

**DOI:** 10.1371/journal.pone.0099131

**Published:** 2014-06-26

**Authors:** Maria Vassilakopoulou, Taiwo Togun, Urania Dafni, Huan Cheng, Jennifer Bordeaux, Veronique M. Neumeister, Mattheos Bobos, George Pentheroudakis, Dimosthenis V. Skarlos, Dimitrios Pectasides, Vassiliki Kotoula, George Fountzilas, David L. Rimm, Amanda Psyrri

**Affiliations:** 1 Yale University, School of Medicine, Department of Pathology, New Haven, Connecticut, United States of America; 2 Yale University, School of Public Health, Department of Biostatistics, New Haven, Connecticut, United States of America; 3 Laboratory of Biostatistics, University of Athens School of Nursing, Athens, Greece; 4 Laboratory of Molecular Oncology, Hellenic Foundation for Cancer Research, Aristotle University of Thessaloniki School of Medicine, Thessaloniki, Greece; 5 Department of Medical Oncology, Ioannina University Hospital, Ioannina, Greece; 6 Second Department of Medical Oncology, “Metropolitan” Hospital, Piraeus, Greece; 7 Oncology Section, Second Department of Internal Medicine, “Hippokration” Hospital, Athens, Greece; 8 Department of Pathology, Aristotle University of Thessaloniki School of Medicine, Thessaloniki, Greece; 9 Department of Medical Oncology, “Papageorgiou” Hospital, Aristotle University of Thessaloniki School of Medicine, Thessaloniki, Greece; 10 Division of Oncology, Second Department of Internal Medicine, University of Athens School of Medicine, Attikon University Hospital, Athens, Greece; Innsbruck Medical University, Austria

## Abstract

**Background:**

We sought to determine the predictive value of in situ mRNA measurement compared to traditional methods on a cohort of trastuzumab-treated metastatic breast cancer patients.

**Methods:**

A tissue microarray composed of 149, classified as HER2-positive, metastatic breast cancers treated with various trastuzumab-containing chemotherapy regimens was constructed. HER2 intracellular domain(ICD), HER2 extracellular domain(ECD) and HER2 mRNA were assessed using AQUA. For HER2 protein evaluation, CB11 was used to measure ICD and SP3 to measure ECD of the HER2 receptor. In addition, HER2 mRNA status was assessed using RNAscope assay ERRB2 probe. Kaplan – Meier estimates were used for depicting time-to-event endpoints. Multivariate Cox regression models with backward elimination were used to assess the performance of markers as predictors of TTP and OS, after adjusting for important covariates.

**Results:**

HER2 mRNA was correlated with ICD HER2, as measured by CB11 HER2, with ECD HER2 as measured by SP3 (Pearson’s Correlation Coefficient, r = 0.66 and 0.51 respectively) and with FISH HER2 (Spearman’s Correlation Coefficient, r = 0.75). All markers, HER2 mRNA, ICD HER2 and ECD HER2, along with FISH HER2, were found prognostic for OS (Log-rank p = 0.007, 0.005, 0.009 and 0.043 respectively), and except for FISH HER2, they were also prognostic for TTP Log-rank p = 0.036, 0.068 and 0.066 respectively) in this trastuzumab- treated cohort. Multivariate analysis showed that in the presence of pre-specified set of prognostic factors, among all biomarkers only ECD HER2, as measured by SP3, is strong prognostic factor for both TTP (HR = 0.54, 95% CI: 0.31–0.93, p = 0.027) and OS (HR = 0.39, 95%CI: 0.22–0.70, p = 0.002).

**Conclusions:**

The expression of HER2 ICD and ECD as well as HER2 mRNA levels was significantly associated with TTP and OS in this trastuzumab-treated metastatic cohort. In situ assessment of HER2 mRNA has the potential to identify breast cancer patients who derive benefit from Trastuzumab treatment.

## Introduction

In recent years, targeted therapies such as the anti-HER2 humanized monoclonal antibody trastuzumab, have changed the therapeutic landscape in breast cancer. *HER2,* a proto-oncogene encoding HER2 tyrosine kinase receptor, is amplified in 10 to 20% of breast cancers, leading to HER2 protein overexpression and an aggressive tumor phenotype associated with reduced survival and high metastatic potential. The advent of molecular targeting of HER2 receptor with trastuzumab has substantially improved the outcome of breast cancer patients. Although single-agent trastuzumab exerts some antitumor activity, the highest clinical benefit is derived when trastuzumab is combined with chemotherapy [1]–[5] HER2 testing has become routine practice in every patient with breast cancer since the benefit of trastuzumab is limited to patients with HER-2 positive breast cancer.

Accurate assessment of HER2 status is necessary to recommend therapy for patients who are most likely to benefit from the treatment and minimize unnecessary overtreatment in the setting where potential side effects may occur [6].

Despite the reported and proven benefits of trastuzumab in HER2-overexpressing metastatic breast cancer patients, approximately 50% of them [7] exhibit de novo resistance while the vast majority of patients who initially respond eventually develop acquired resistance within a year [8].

The assessment of HER2 overexpression by two immunohistochemical (IHC) assays and three fluorescent in-situ hybridization (FISH) assays have been approved by the US Food and Drug Administration (FDA) [9]–[13] However, these methods are suboptimal since up to 33% of patients will not respond to trastuzumab despite their tumors meeting the HER2 prerequisite as determined by these methods [8]. Moreover, recent studies suggest that some patients who are not classified as HER2 positive may benefit from trastuzumab [14]. Differences in methodology and scoring systems have led to varying results in different studies and patient cohorts, contributing to the debate on the optimal testing method and the role of HER2 as a prognostic and predictive factor. Two independent cooperative group studies reported a less than optimal concordance between locally and centrally performed HER2 assays, as up to 20% of locally performed HER2 assays could not be confirmed by central laboratories [15].

To minimize discrepancies, the American Society of Clinical Oncology (ASCO) and College of American Pathologists (CAP), developed guidelines for optimal laboratory evaluation of HER2 status by modifying the FDA criteria, which had been used in pivotal trastuzumab trials [16]. However, data analysis from the phase III N9831 trial that investigated adjuvant trastuzumab therapy (NCCTG N9831 Clinical Trial Registration. National Institutes of Health Clinical Trials Website. http://clinicaltrials.gov/ct2/show/NCT00005970?term=N9831&rank=2. Accessed April 18, 2011), showed decrease in the number of patients eligible for trastuzumab therapy when the ASCO/CAP criteria were applied [17]. This analysis showed that the adoption of ASCO/CAP criteria may be too restrictive, increasing the “false negative cases” and may disallow up to 4% of patients from receiving anti-HER2 therapy. As a result, ASCO/CAP criteria may exclude patients who would have been eligible for the trastuzumab in the pivotal clinical trials, which led to its approval. Hence, ASCO/CAP has recently revised their guidelines to the original 10% cutpoint [18].

In spite of these changes, efforts to optimize testing methods are ongoing. It has been suggested that HER2 status can be assessed with different approaches as a continuous variable and can be assessed on mRNA level [19]. HER2 gene amplification determined with FISH is strongly associated with elevated mRNA and protein levels and small studies have reported on the concordance of HER2 status by RT-PCR to that by IHC and FISH [20]–[22]; Two independent studies have reported on the concordance between HER2 Quantitative Reverse Transcription Polymerase Chain Reaction (qRT-PCR) of the Oncotype Dx Test and the FDA-approved IHC/FISH assays and have yielded conflicting results [23], [24]. In the first study, a greater than 50% false-negative rate for Onco*type* DX RT-PCR for *HER2* assessment was reported. This high false-negative rate of RT-PCR assay highlights the shortcomings of this non-morphologic assay and the importance of standard immunohistological methods in HER2 testing. This result could open the door for a new method of in situ quantitative analysis of HER2 mRNA levels that would reflect more precisely HER2 status by combining quantitative determination of gene expression levels and morphologic assessment.

The aim of this study was to assess HER2- mRNA as a potential predictor of benefit from trastuzumab-based chemotherapy and to correlate it to HER2 protein levels and to FISH by using a combination of automated-quantitative immunofluorescence and a new method of mRNA in situ hybridization marketed as RNAscope.

## Materials and Methods

### Patient cohort

The breast cancer cohort used for this study consists of 149 patients classified as HER2-overexpressing by locally performed IHC at the time of pathological assessment, who were diagnosed in Greece from 1999 through 2006 with metastatic breast cancer. Patients were treated with various Trastuzumab-based combinations for metastatic disease [25], [26]. Tissue samples were collected prior to treatment and none of the patients received neoadjuvant therapy. A tissue microarray (TMA) was constructed, composed by histospots of the above cohort. Each clinical case was represented by 2 cores on this TMA (2-fold redundant). All cases were centrally reviewed for HER2 overexpression/amplification by IHC and FISH at the Laboratory of Molecular Oncology, Hellenic Foundation for Cancer Research, Aristotle University of Thessaloniki School of Medicine. Clinical data were comprehensively obtained after review of medical records. Clinicopathologic characteristics of the cohort are found in Table 1. Only patients receiving Trastuzumab as 1^st^ line (n = 130) were included in the analysis. The translational research protocol was approved by the Bioethics Committee of the Aristotle University of Thessaloniki School of Medicine (Protocol #4283; January 14, 2008) under the general title “Investigation of major mechanisms of resistance to treatment with trastuzumab in patients with metastatic breast cancer”. All patients included in the study after 2005 provided written informed consent for the provision of biological material for future research studies, before receiving any treatment. Waiver of consent was obtained from the Bioethics Committee for patients included in the study before 2005.

**Table 1:** Cohort Description.

Variables		Number	%
**Age**			
	<50	39	26%
	>50	110	74%
**Grade**			
	1	3	2%
	2	53	36%
	3	83	56%
	Unknown	10	6%
**Distant Metastasis**			
	Yes	132	89%
	No	11	7%
	Unknown	6	4%
**ER**			
	Positive	104	70%
	Negative	45	30%
**PR**			
	Positive	76	51%
	Negative	73	49%
**HER2**			
	Positive	90	60%
	Negative	59	40%
**Trastuzumab**			
1st Line		130	
	Monotherapy	4	3%
	with Anthracycline	24	19%
	with Taxane	102	78%
2nd Line +		19	
	Monotherapy	3	16%
	with Anthracycline	4	21%
	with Taxane	12	63%

### RNA in situ hybridization

The RNAscope (Advanced Cell Diagnostics, Inc., Hayward, CA [ACD]) technique of mRNA *in situ* hybridization (ISH) formalin-fixed paraffin embedded (FFPE) tissue has been previously described [27].

Briefly, the assay uses a pool of up to 20 probe pair sets for each mRNA target of interest. Probe pairs bind along an mRNA region and create a unique 28 base-pair sequence recognized by the preAMP which then allows for binding during the subsequent amplification steps and finally the amplified target is detected by cy5 tyramide & AQUA (Figure S1).

HER2 mRNA status was assessed by in situ hybridization using the RNAscope FFPE assay kit according to the manufacturer’s instructions modified for fluorescence detection of transcripts using Cy5-tyramide. In brief, slides with TMA sections were treated with heat and protease digestion followed by hybridization with target probes to *ERBB2 gene (by ACD)*, the housekeeping gene *ubiquitin C* (*UbC*) as a positive control or the bacterial gene *DapB* as a negative control. *ERBB2 gene* or *UbC* specific hybridization signals were detected with Cy5-tyramide. Sections were then incubated with 0.3% bovine serum albumin (BSA) in 0.1 mol/L of Tris-buffered saline (triethanolamine-buffered saline, pH 8) for 30 minutes at room temperature followed by incubation with a wide-spectrum rabbit anti-cow cytokeratin antibody (Z0622 1∶100, DAKO) in BSA/tris-buffered saline for 1 hour at room temperature. The cytokeratin signal was detected with Alexa 546 conjugated goat anti-rabbit (1∶100, Molecular Probes) incubated for 1 hour at room temperature. Slides were then mounted using ProlongGold plus 4,6-diamidino-2-phenylindole (DAPI).

### Immunofluorescence staining

In Situ quantitative measurement of biomarkers was done by using the following:

- anti-HER2 mouse monoclonal antibody, clone CB11 (by Biocare) Epitope: Intracellular domain of human HER2 receptor.
- anti-HER2 mouse monoclonal antibody, clone SP3 (by Thermo Fischer) Epitope: Extracellular domain of human c-erbB2.
- ERRB2 probe, for HER2 mRNA (RNAscope assay by ACD), according to the manufacturer’s protocol modified for detection with Cy-5 Tyramide.

Each antibody was validated by performing 1) titering, 2) reproducibility assessment on index arrays, and 3) verification of linearity with expression on cell line series, according to a previously described protocol [28]. The immunofluorescence staining was performed by using a standard protocol. In brief, TMA slides were deparaffinized with xylene and rehydrated with ethanol. Antigen retrieval was performed with citrate buffer (pH = 6) at 97°C for 20 minutes. In order to block endogenous peroxidase activity, we used a 2.5% solution of hydroxyl peroxide in methanol, and thereafter slides were incubated with the primary antibody and cytokeratin (Cytokeratin (KRT X) Mouse AE1/AE3/IgG1 M3515 DAKO, Rabbit polyclonal ZO622 DAKO) overnight at 4°C. Each staining was performed by using the Thermo/Fisher Lab Vision autostainer. As secondary antibody we used Alexa 546 conjugated goat antirabbit/mouse (Molecular Probes, Eugene OR) with Mouse/rabbit EnVision reagent (DAKO) and sequentially Cy5-tyramide (Perker Elmer, Life Science, MA). DAPI was used to stain the cell nuclei.

### Quantitative immunofluorescence (QIF)

The AQUA method of QIF has been described elsewhere [29]. This method allows exact and objective measurement of fluorescence intensity within a defined tumor area, as well as within subcellular compartments. Briefly, a series of high-resolution monochromatic images were captured using an Olympus AX-51 epifluorescent microscope based on a previously described algorithm for image collection [29]. According to this algorithm, images were obtained for each sample histospot and for each different fluorescence channel, DAPI (nuclei), Alexa 546 (cytokeratin), or Cy5 (target probe), respectively. A tumor mask was created by binarizing the cytokeratin signal to distinguish stromal from tumor area and target probe expression was quantified only in the tumor. AQUA scores were calculated for a given target within the “tumor mask” by dividing the signal intensity by the area of the “tumor mask” within each histospot. Histospots containing less than 5% tumor, as determined by the percentage of area which was positive for cytokeratin were excluded from the analysis.

### Statistical analysis

Correlation of each biomarker with immunohistochemistry/FISH was assessed by using the Pearson and Spearman’s rank correlation coefficient. All biomarkers were treated as binary variables. Dichotomization of CB11 and SP3 was based on the corresponding median AQUA scores (620.41 and 99.42 respectively), whilst the signal-to-noise threshold was used as a cut point for HER2mRNA (<100: negative vs. ≥100 positive). Overall survival (OS) and time to progression (TTP) were the primary endpoints of interest. TTP survival times were calculated in months, from Trastuzumab initiation to the date of disease progression, censoring or last follow-up exam. Survival curves were calculated using the Kaplan-Meier method and differences in survival times between groups were assessed by using the log-rank test. Multivariate Cox regression models were used to assess the performance of each marker and FISH HER2 after adjusting for other important predictors, namely age group (<50 vs. ≥50 years), disease grade (I & II vs. III), status of distant metastasis and ER status. For a simultaneous assessment of all HER2 biomarkers and FISH in the presence of the aforementioned pre-specified set of prognostic factors, multivariate Cox regression models, with backward selection, were also fitted. During the backward elimination process, the possibility of significant interactions between any pair of biomarkers and each biomarker by ER status was also tested. None of these candidates was found statistically significant and hence no interaction terms are included in the final multivariate Cox proportional hazards (PH) models.

Statistical analysis was performed using R Statistical Software (Foundation for Statistical Computing, Vienna, Austria, http://www.r-project.org/).

## Results

### Correlation between HER2 mRNA, HER2- protein and HER2-FISH

**Figure 1:**
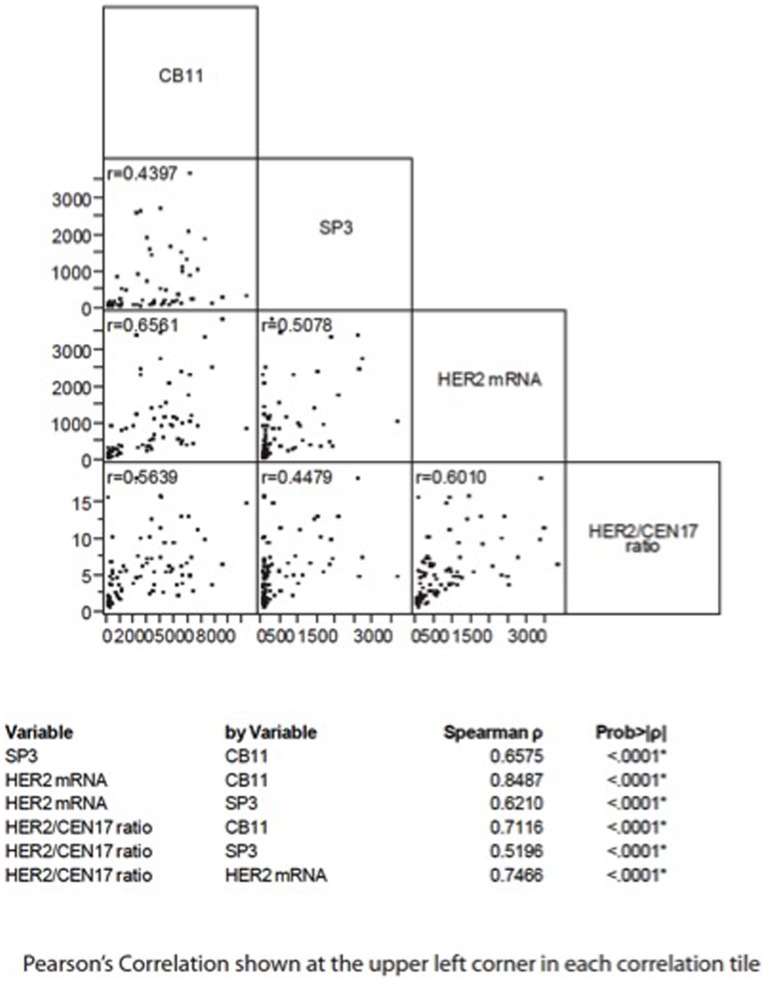
Pearson’s Correlation of CB11, SP3, Her2mRNA and FISH.

The quantitative ISH assay allows for comparison between the HER2 mRNA and protein levels, both of which are quantified on a continuous scale using AQUA on serial TMA sections from the breast cancer cohort. Our cohort demonstrated a positive, linear correlation between HER2 mRNA and protein levels. HER2 mRNA was correlated with ICD HER2, as measured by CB11, with ECD HER2 as measured by SP3 (Pearson’s r = 0.66 and 0.51 respectively) and with FISH HER2 (Spearman r = 0.75, Fig. 1).

**Figure 2:**
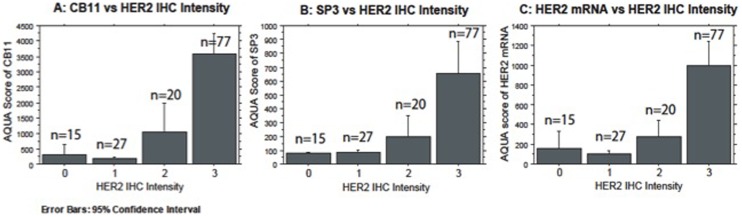
Comparison of CB11 (A), SP3 (B), Her2mRNA (C) to Her2 IHC.

Additionally, HER-2 ICD, ECD and HER2 mRNA AQUA scores showed a significant correlation with immunohistochemistry as centrally tested (Fig. 2A, B and C, respectively).

### Time To Progression (TTP)

**Figure 3:**
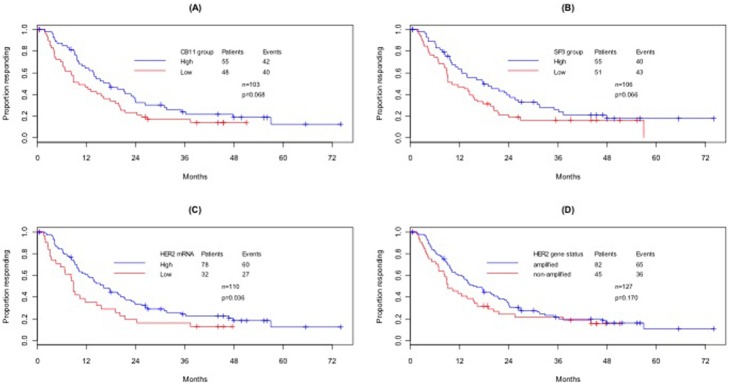
Time to progression (TTP) by HER2 patient populations, as defined by each biomarker and HER2 gene status (Kaplan-Meier plots). (A) ICD HER2 as measured by CB11 (high vs. low); (B) ECD HER2 as measured by SP3 (high vs. low); (C) HER2 mRNA (high vs. low); (D) FISH HER2 (amplified vs. non-amplified).

Similarly, patients with high HER2 ICD as well as ECD protein expression showed a longer TTP survival compared to patients with low protein expression levels (Log-rank p = 0.068 and 0.064 respectively, Fig. 3A and 3B) as determined by the median AQUA score for each marker. Kaplan-Meier analysis showed a longer TTP survival of patients with high HER2 mRNA compared to patients with low HER2 mRNA (Log-rank p  = 0.036, Fig. 3C) as determined by the detection threshold (the highest noise level measured by DapB as negative control). No significant difference was found between patients with amplified and non-amplified HER2 gene status (Log-rank p = 0.170, Fig. 3D).

Cox proportional hazard models fitted for each biomarker separately showed that for given age group, disease grade, status of distant metastasis and ER status, TTP survival is more favorable for HER2 high patients, as defined by the corresponding median AQUA scores. The HR estimated by these models were HR  = 0.52 (95%CI: 0.31–0.88, p = 0.014) for CB11, HR = 0.46 (95%CI: 0.27–0.48, p = 0.004) for SP3 and HR = 1.68 (95%CI: 1.00–2.83, p = 0.051) for HER2 mRNA. In a similar model with all pre-specified prognostic factors present, TTP survival was also more favorable for patients with amplified HER2 gene status (HR = 0.56, 95%CI: 0.34–0.91, p = 0.018).

The multivariate TTP analysis included all biomarkers and FISH HER2 as well as the aforementioned prognostic factors. Applying a backward elimination process, it was found that ECD HER2, as measured by SP3, is the only biomarker that retains its prognostic ability for TTP survival (HR = 0.54, 95%CI: 0.31–0.93, p = 0.027). FISH HER2 was also marginally significant at α = 10% (Table 2).

**Table 2:** TTP analysis: Predictive evaluation of HER2 biomarkers and FISH for given prognostic factors (Cox Proportional Hazards Model).

	Hazard ratio (HR; 95% CI)	P-value
SP3 (High vs. Low)	0.54 (0.31, 0.93)	0.027
HER2 gene status (amplified vs. non-amplified)	0.62 (0.35, 1.10)	0.101
Age group (<50 vs. > = 50)	0.79 (0.46, 1.38)	0.417
Disease grade (I & II vs. III)	0.84 (0.51, 1.37)	0.483
Distant metastasis (no vs. yes)	0.24 (0.07, 0.80)	0.020
ER status (negative vs. positive)	1.96 (1.09, 3.51)	0.024

#### Overall Survival (OS)

**Figure 4:**
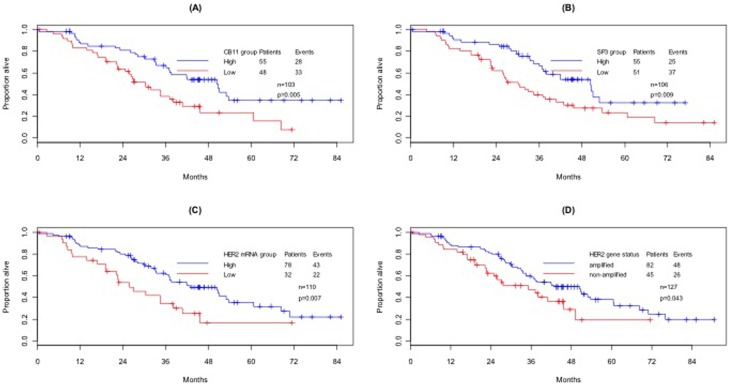
Overall Survival (OS) by HER2 patient populations, as defined by each biomarker and HER2 gene status (Kaplan-Meier plots). (A) ICD HER2 as measured by CB11 (high vs. low); (B) ECD HER2 as measured by SP3 (high vs. low); (C) HER2 mRNA (high vs. low); (D) FISH HER2 (amplified vs. non-amplified).

OS was found significantly longer for patients with high HER2 ICD (Log-rank p = 0.005, Fig. 4A), high ECD protein expression (Log-rank p = 0.009, Fig. 4B), high HER2 mRNA (Log-rank p = 0.007, Fig. 4C) and amplified HER2 gene status (Log-rank p = 0.043, Fig. 4D).

Separate Cox PH models for each HER2 biomarker and FISH showed that for a given age group, grade of disease, ER status and status of distant metastasis, OS is more favorable for patients with high HER2 ICD (HR = 0.38, 95%CI: 0.21–0.68, p = 0.001), high HER2 ECD (HR = 0.39, 95%CI: 0.22–070, p = 0.002), high HER2 mRNA (HR = 0.46, 95%CI: 0.26–0.81, p = 0.007) and amplified HER2 gene status (HR = 0.42, 95%CI: 0.24–0.72, p = 0.002).

Multivariate analysis showed that after adjustment for the pre-specified set of prognostic factors, high HER2 ECD protein levels, as measured by SP3, predict better overall survival (HR = 0.39, 95%CI: 0.22–070, p = 0.002, Table 3). None of the remaining biomarkers was found statistically significant in the multivariate model.

**Table 3:** OS analysis: Predictive evaluation of HER2 biomarkers and FISH for given prognostic factors (Cox Proportional Hazards Model).

	Hazard ratio (HR; 95% CI)	P-value
SP3 (High vs. Low)	0.39 (0.22, 0.70)	0.002
Age group (<50 vs. > = 50)	0.91 (0.49, 1.68)	0.755
Disease grade (I & II vs. III)	0.56 (0.32, 1.00)	0.051
Distant metastasis (no vs. yes)	0.25 (0.06, 1.08)	0.063
ER status (negative vs. positive)	1.50 (0.81, 2.77)	0.196

## Discussion

Analyzing biomarkers at the mRNA level for prognostic classification of patients has become popular in recent years. As an example, mRNA analysis is the basis of the FDA-cleared Agendia’s MammaPrint test [30] which measures the mRNA level of 70 specific genes in fresh tissue and Genomic Health’s popular Oncotype Dx test [31] which uses a RTQ-PCR process to quantify the expression of specific mRNA for 21 cancer genes in FFPE material, both tests for assessing recurrence risk.

It has been previously shown that both HER2 protein overexpression and gene amplification are closely correlated with mRNA levels in formalin-fixed, paraffin-embedded tissue sections, especially when tumour tissue is microdissected [21], [32]. The novelty of our study lies on determination of HER2 mRNA levels using a method of quantitative in situ RNA assessment.

The HER2 status is tested on all newly diagnosed breast cancer cases as prognostic factor and predictor for anti-HER2 targeted therapy [33]. The therapeutic response to anti-HER2 treatment can be predicted by HER2 status and is associated with improved outcome in patients with metastatic and operable HER2-positive breast cancers [34]–[36]. Consistent with these findings, we showed that HER2-protein levels and HER2-m RNA expression are predictors of TTP after trastuzumab-containing chemotherapy in this metastatic breast cancer cohort.

We showed that HER2 ECD as assessed by the SP3 antibody trends to outperform the rest of the HER2 biomarkers and FISH as a predictor for median TTP after trastuzumab therapy. SP3 antibody has been compared to the CB11 antibody (used in the FDA-approved PATHWAY kit-Ventana), Herceptest and FISH and showed a higher discrimination power compared to HercepTest [37]–[39] while it showed a high concordance with FISH [40].

One possible explanation of our findings may reside in the molecular target of trastuzumab which is the extracellular domain of HER-2 [41] which may be cleaved and shed from the surface of breast cancer cells generating a truncated 95-kd intramembrane protein [42]. This truncated form of HER2 does not possess the extracellular trastuzumab-binding epitope, and its expression has been associated with trastuzumab resistance [43]. Hence, the assessment of the HER2-ECD may be of particular interest and predictive significance as the actual target epitope of trastuzumab.

Our study also shows a significant correlation of HER2-mRNA levels with conventional immunohistochemistry and in situ-hybridization methods as well as FISH. We found that HER2 status by FISH, AQUA HER2-proteins and HER2-mRNA levels are all independent predictor factors for TTP after Trastuzumab-containing chemotherapy in this metastatic cohort. In our study, HER2 mRNA status was assessed by in situ hybridization using the RNAscope FFPE assay in combination with the AQUA method of automated quantitative immunofluorescence. In situ quantitative measurement of both HER2-mRNA and protein; is reproducible, automated method that reduces intra-observer variability. Since the interpretation of HER2 immunostaining and in situ-hybridization may be influenced by laboratory and observer variability, the use of the AQUA automated method in measurement of HER2 protein along with the HER2-mRNA level could improve the diagnostic accuracy of HER2 status,. QISH enables a relatively easy, fast and reproducible quantification of HER2-mRNA expression feasible in routine FFPE tissue.

In summary, our study demonstrates that measurement of HER2-m RNA levels with this novel method has a predictive value for response to Herceptin-based chemotherapy in metastatic breast cancer. Additional studies in prospective cohorts are required to validate these findings with the ultimate goal to build a potential predictive model of trastuzumab therapy and complete the puzzle of HER2 testing optimization.

## Supporting Information

Figure S1 He2mRNA hybridization.
(TIF)
