# Supplementary figures and images for: In Situ Quantitative Measurement of HER2mRNA Predicts Benefit from Trastuzumab-Containing Chemotherapy in a Cohort of Metastatic Breast Cancer Patients
Source: PLoS One. 2014 Jun 26;9(6):e99131. doi: 10.1371/journal.pone.0099131 (PMC4072595; doi:10.1371/journal.pone.0099131)

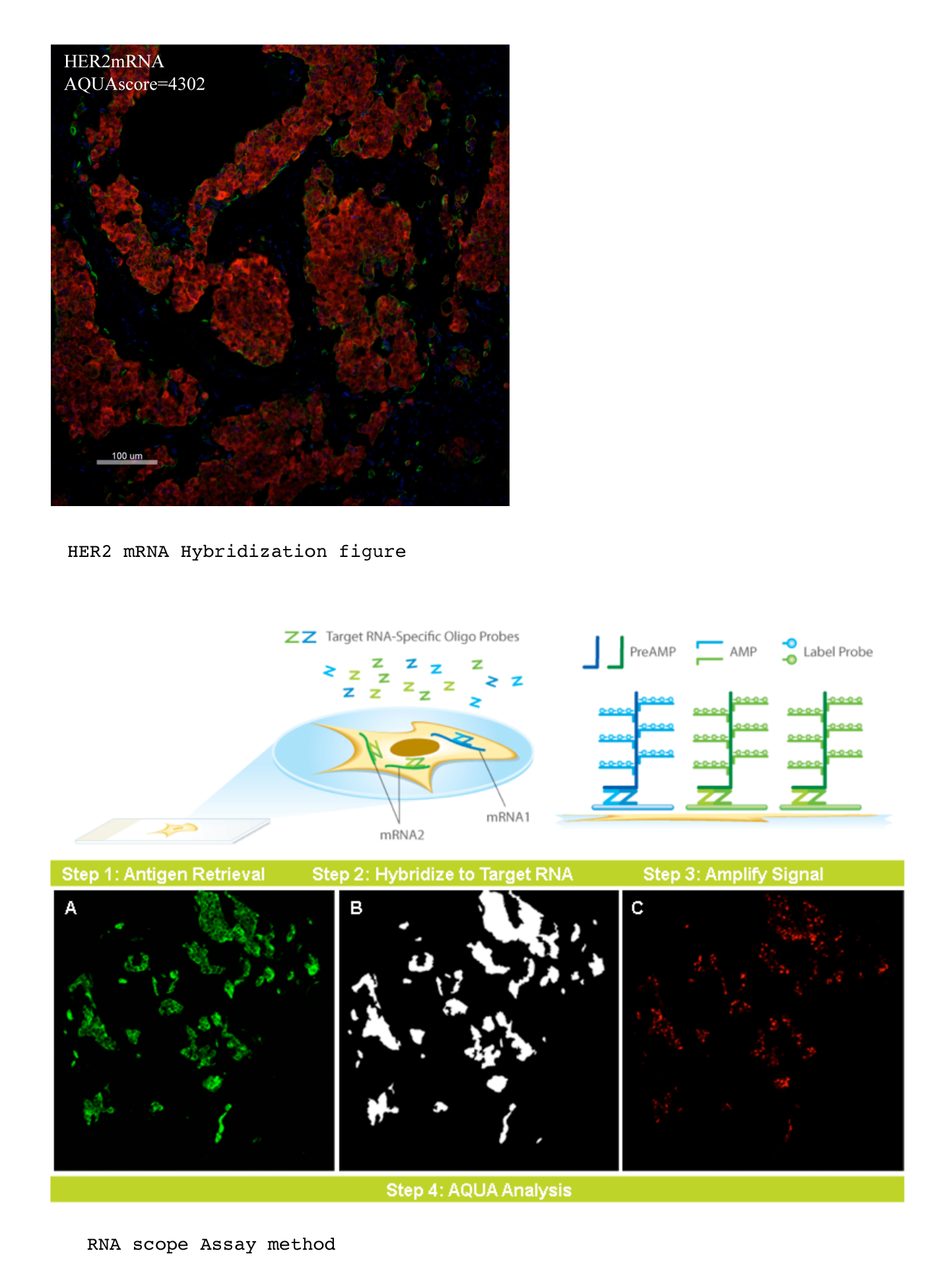

Supplement: Figure S1 — He2mRNA hybridization. (TIF) [file pone.0099131.s001.tif]
